# A Retrospect of a Second Series of Fifty Consecutive Intra-Abdominal Operations

**Published:** 1900-06

**Authors:** James Swain

**Affiliations:** Professor of Surgery at University College, Bristol; Assistant Surgeon to the Bristol Royal Infirmary


### A RETROSPECT OF A SECOND SERIES OF FIFTY CONSECUTIVE INTRA-ABDOMINAL OPERATIONS.

BY

JAMES SWAIN, M.S., M.D. Lond., F.R.C.S. Eng.,

*Professor of Surgery at University College, Bristol; Assistant Surgeon to the Bristol Royal Infirmary.*

---

As in the first series,[1] the term "intra-abdominal operation" is used in order that such operations as herniotomy, vaginal hysterectomy, &c. (which rarely require intra-abdominal manipulation of the kind here meant), may be excluded.

[1] *Bristol M.-Chir. J.*, 1898, xvi. 211.

| No. | Sex and Age. | Medical Attendant. | Disease. | Treatment. | Result. |
|---|---|---|---|---|---|
| 1 | F. 44 | ... | Peritoneal adhesions | Adhesions freed | Recovered. |
| 2 | M. 56 | ... | Malignant stricture of rectum | Sigmoidostomy | Death. |
| 3 | F. 19 | ... | Malignant growth in pelvis | Exploratory cœliotomy | Recovered. |
| 4 | F. 16 | Dr. J. Young | Appendicitis—diffuse peritonitis | Drainage | Death. |
| 5 | F. 37 | Mr. F. N. Lewarne | Ovarian adeno-cystoma (left); multiple cysts (right) | Ovariotomy (left); removal of right ovary and tube | Recovered. |
| 6 | M. 51 | Dr. E. L. Lees | Malignant disease of ascending colon | Exploratory cœliotomy | Recovered. |
| 7 | F. 47 | Dr. J. C. Maclean | Retro-peritoneal lipoma | Exploratory cœliotomy | Recovered. |
| 8 | M. 33 | Dr. M. G. Dobbyn | Infective cholangitis; portal pyæmia | Cholecystostomy | Relieved. |
| 9 | F. 24 | Mr. D. H. Forty | Appendicitis | Appendicectomy | Recovered. |
| 10 | F. 38 | Dr. W. E Pountney | Ovarian adeno-cystoma (left) | Ovariotomy | Death. |
| 11 | F. 58 | Mr. F. Dymoke | Ovarian adeno-cystoma (left) | Ovariotomy | Recovered. |
| 12 | F. 31 | Dr. J. C. Maclean | Pyo-salpinx (double) | Salpingo-oöphorectomy (double) | Recovered. |
| 13 | F. 38 | Mr. L. H. Williams | Pelvic abscess. ? Ruptured tubo-ovarian abscess | Drainage | Recovered. |
| 14 | M. 10 | Dr. W. Brown | Appendicitis (gangrenous) | Appendicectomy | Death. |
| 15 | M. 62 | Dr. W. McD. Ellis | Malignant stricture of ascending colon | "Short circuiting" (ileo-colostomy) | Relieved. |
| 16 | M. 40 | Mr. D. H. Forty | Appendicitis | Appendicectomy | Recovered. |
| 17 | M. 6 | Mr. D. H. Forty | Appendicitis | Drainage | Recovered. |
| 18 | F. 6 | Dr. E. L. Lees | Tubercular peritonitis | Cœliotomy—curettage | Recovered. |
| 19 | M. 43 | Mr. R. W. Statham | Intestinal obstruction; malignant stricture of sigmoid flexure | Sigmoidostomy; colectomy | Recovered. |
| 20 | F. 33 | ... | Salpingo-oöphoritis | Salpingo-oöphorectomy (double) | Recovered. |
| 21 | M. 46 | Mr. H. T. Rudge | Cholæmia; malignant disease of pancreas | Cholecystostomy | Death. |
| 22 | F. 63 | Mr. A. H. Haines | Acute cholecystitis; cholelithiasis | Cholecystostomy | Recovered. |
| 23 | F. 33 | Dr. H. S. McQuade | Carcinoma of pylorus | Exploratory cœliotomy | Recovered. |
| 24 | F. 33 | Dr. J. C. Maclean | Ovarian adeno-cystoma (left); multiple cysts (right) | Ovariotomy (left); removal of right ovary and tube | Recovered. |

| | | | | | |
|---|---|---|---|---|---|
| 25 | F. 36 | Dr. J. C. Maclean ... | Myo-fibroma of uterus ... ... ... | Retro-peritoneal hystero-myomectomy | Recovered. |
| 26 | M. 49 | Mr. R. Kinneir ... | Cancerous stricture of descending colon | Colectomy ... ... ... ... | Death. |
| 27 | F. 29 | Dr. R. R. Davidson... | Broad ligament cyst (right); cystic ovary (right) | Enucleation of cyst; salpingo-oöphorectomy | Recovered. |
| 28 | F. 51 | Dr. E. Markham Skerritt | Appendicitis ... ... ... ... | Appendicectomy ... ... ... | Death. |
| 29 | F. 20 | Dr. H. S. McQuade... | Ovarian adeno-cystoma (left) ... ... | Ovariotomy ... ... ... ... | Recovered. |
| 30 | F. 25 | Mr. F. S. Cowan ... | Appendicitis; tubercular mesenteric glands | Appendicectomy; removal of glands | Recovered. |
| 31 | F. 49 | Mr. W. Danne ... | Ovarian adeno-cystoma (right); multiple cysts (left) | Ovariotomy (right); salpingo-oöphorectomy (left) | Recovered. |
| 32 | F. 25 | Dr. H. Skelton ... | Appendicitis ... ... ... ... | Appendicectomy ... ... ... | Recovered. |
| 33 | M. 48 | Dr. C. E. Wigan ... | Intestinal obstruction ... ... ... | Enterostomy ... ... ... ... | Death. |
| 34 | F. 30 | Dr. J. C Maclean ... | Ovarian adeno-cystoma (left) ... ... | Ovariotomy ... ... ... ... | Recovered. |
| 35 | F. 50 | Dr. H. L. Ormerod... | Carcinoma of peritoneum ... ... | Exploratory cœliotomy ... ... | Recovered. |
| 36 | M. 62 | Dr. H. Skelton ... | Cancer of rectum ... ... ... | Sigmoidostomy ... ... ... ... | Recovered. |
| 37 | F. 34 | ... ... ... ... | Dermoid of ovary (left) ... ... ... | Ovariotomy ... ... ... ... | Recovered. |
| 38 | M. 73 | Dr. E. Markham Skerritt | Cancer of rectum; intestinal obstruction | Sigmoidostomy ... ... ... ... | Death. |
| 39 | F. 44 | ... ... ... ... | Myo-fibroma of uterus ... ... ... | Retro-peritoneal hystero-myomectomy | Recovered. |
| 40 | F. 56 | Mr. G. M. Swinhoe... | Hydrops of gall-bladder ... ... | Cholecystostomy ... ... ... | Recovered. |
| 41 | F. 56 | Mr. E. F. Clowes ... | Ovarian adeno-cystoma (left) ... ... | Ovariotomy ... ... ... ... | Recovered. |
| 42 | M. 12 | Dr. J. C. Maclean ... | Appendicitis ... ... ... ... | Appendicectomy ... ... ... | Recovered. |
| 43 | F. 60 | Dr. W. A. Lauder Smith | Carcinoma of ascending colon ... | Colectomy ... ... ... ... | Death. |
| 44 | F. 32 | Dr. J. Ambrose ... | Appendicitis ... ... ... ... | Drainage ... ... ... ... ... | Recovered. |
| 45 | M. 42 | Dr. E. F. Martin ... | Appendicitis ... ... ... ... | Appendicectomy ... ... ... | Recovered. |
| 46 | F 59 | ... ... ... ... | Malignant stricture of descending colon | Colostomy ... ... ... ... | Death. |
| 47 | F. 15 | Dr. H. S. McQuade... | Ovarian dermoid (left): ovarian adeno-cystoma (left) | Ovariotomy ... ... ... ... | Recovered |
| 48 | M. 58 | Dr. S. Skinner ... | Vesico-intestinal fistula; intestinal obstruction | Enterostomy ... ... ... ... | Death. |
| 49 | M. 25 | Dr. H. J. Doidge ... | Hepatic abscess ... ... ... ... | Trans-pleural hepatotomy ... ... | Recovered. |
| 50 | M. 45 | Mr. W. Thompson ... | ? Intestinal obstruction ... ... ... | Exploratory cœliotomy ... ... | Recovered. |

This series is continuous with the last, and contains the usual diversity of cases met with in abdominal surgery.

The mortality of cases of intestinal obstruction, including malignant disease of the intestine, is unnecessarily high. Every one now recognises the desirability of early operation in strangulated external hernia, and yet in acute internal obstructions—where the pathological results are more or less identical with those obtaining in external hernia—there is too often a tendency to delay operation until the chances of success of operative interference have been reduced to a minimum, and the patient pays the penalty.

The difficulty in diagnosis is, doubtless, largely answerable for this deplorable hesitancy in necessary treatment, but I cannot emphasise too strongly the duty of urging an exploratory cœliotomy (for the actual cause of obstruction is frequently undiscoverable before operation) directly the diagnosis of acute intestinal obstruction is established or is reasonably suspected to exist. In "late" cases the patient does not die from operation, but from the previous absorption of the accumulated poisonous products of his own intestines.

Much the same remarks might be made with regard to the more chronic forms of obstruction usually associated with malignant disease. The operations of sigmoidostomy, ileo-colostomy, &c., will considerably diminish in number when it becomes more widely recognised that by early operation alone can we hope to freely remove malignant growths of the intestine, which, under such favourable circumstances, show less tendency to recurrence than malignant disease in many parts of the body. A radical operation (enterectomy, &c.), performed in an early stage, may not only rid the patient of his disease for ever, but is far less dangerous to life than a palliative operation (ileo-colostomy, &c.) which has been deferred until the vital functions have been reduced to a low ebb by constant vomiting, continued pain, and other results of the absorption of the products of intestinal putrefaction and stoppage of the circulation of the contents of the intestines.

The tabular list of cases (pp. 104 and 105) is a chronological one, and, therefore, no attempt has been made at classification.

In discussing these cases, those of a similar nature will be grouped together; but details will only be given in the more important ones, and comment made where desirable.

A.—**Operations for Diseases of the Ovaries and Fallopian Tubes.** Cases 5, 10, 11, 12, 13, 20, 24, 27, 29, 31, 34, 37, 41, and 47.

Clinically, these cases may be divided into—

**Adeno-cystomata.**—Cases 5, 10, 11, 24, 29, 31, 34, 41, and 47.
**Intra-ligamentary cyst.**—Case 27.
**Salpingo-oöphoritis.**—Cases 12, 13, and 20.
**Dermoid tumours.**—Cases 37 and 47.

The **adeno-cystomata** call for no special comment, but it is noticeable that in a large proportion of the cases the opposite ovary was so far degenerated as to require removal. A wise conservatism should however be practised whenever possible, and in young women it is desirable that any considerable portion of healthy ovarian tissue should be preserved by the adoption of a resection rather than a complete removal of the organ.

**Intra-ligamentary cysts** are doubtless usually produced by the growth of ovarian tumours between the layers of the broad ligament. The case to be presently narrated, in which the cyst in the ovary appeared to be quite distinct from the intra-ligamentary cyst, would suggest that in some cases the origin of these tumours is not always so easily explained. These tumours are closely applied to the uterus, and they possess no pedicle as in the ordinary ovarian adeno-cystoma. Their removal is frequently attended with difficulty, but the necessary manipulations are much facilitated by the adoption of the Trendelenburg posture. The method of operation is sufficiently indicated in the following description :—

Case 27.—**Intra-ligamentary cyst**—**Enucleation.**—Two years ago the patient was ill for a fortnight with pain in the right side of the abdomen, fever and vomiting. A large swelling was noticed in the right iliac region, and the case was regarded as one of intra-peritoneal hæmatocele. The swelling became rapidly absorbed. A similar attack occurred five months ago, and since then there had been almost continuous pain on the right side of the abdomen. On examination, I found the uterus retroverted, not enlarged, but with some impairment of its mobility. On the right of the uterus, and closely connected with it, was a smooth globular cystic swelling about the size of a hen's egg, occupying the broad ligament, and felt bimanually to reach well above Poupart's ligament. The tumour appeared fixed by infiltration in the broad ligament. A median incision was made above the pubes. On opening the peritoneum considerable matting was found on the right side of the pelvis, and several bands of omentum—attached to the uterus, ovary, etc.—were tied off. The right ovary was very adherent and cystic, and in breaking down some adhesions the cyst in the ovary burst and a chocolate-coloured fluid escaped. The ovarian vessels were then ligatured near the pelvic brim, and the broad ligament opened up by a transverse incision over the top of the cyst within it. The intra-ligamentary cyst was then enucleated by gradually separating the surrounding broad ligament with the fingers. In doing this the cyst burst and a clear fluid escaped. A ligature was then placed near the right cornu of the uterus, so as to include the utero-ovarian anastomosis, and the collapsed intra-ligamentous and ovarian cysts and the Fallopian tube were then cut away. No drainage was employed. The patient made a speedy recovery.

The performance of **salpingo-oöphorectomy** in cases of chronic tubal and ovarian inflammation, pyosalpinx, &c., is often one of great difficulty from the complexity of the adhesions. In some of these cases it is easier to remove the uterus with the appendages (hystero-salpingo-oöphorectomy) than the appendages only, though this was not necessary in Cases 12 and 20. The Trendelenburg position is most useful in dealing with the firm adhesions existing in these cases. The pus in many cases of **pyosalpinx** is fortunately sterile, as was found in Case 12. It is interesting to notice that menstruation continued to be regular in Cases 12 and 20, in spite of the removal of the appendages.

**Vaginal drainage** has a wide field of application in "pelvic abscess" in the female, whether the abscess is a result of a burst tubo-ovarian abscess, appendicitis, or other cause. In cases such as the following, where an abdominal section discloses the existence of a large abscess burrowing deeply among the pelvic viscera, I cannot speak too highly of its value, for I believe its adoption has resulted in the saving of lives that would have been lost by abdominal drainage alone.

CASE 13.—**Ruptured tubo-ovarian abscess—Abdomino-vaginal drainage.**—The patient had attacks of pelvic peritonitis, eleven, four, and two years ago respectively. Three weeks before I saw her a rigor occurred, accompanied by considerable lumbar pain, followed in nine days by sudden collapse, great hypogastric pain and abdominal distension. The temperature varied from 99° to 103°. The day before I saw her there was further collapse and pain, and the abdominal distension increased. There was dorsal decubitus, with the legs drawn up; the expression was one of great anxiety; the tongue was furred and dry, and the pulse very soft. The abdomen was greatly distended, and universally tympanitic. Tenderness was general over the abdomen, but more marked in the hypogastrium. *Per vaginam*, the uterus was embedded in a hard inflammatory mass, which occupied the whole pelvic floor, and no soft spot could be felt. A median incision was made half-way between the umbilicus and pubes. On opening the peritoneum, a good deal of free turbid fluid escaped. Coils of intestine in the hypogastrium and pelvis were matted together by soft adhesions. As the adhesions were separated loculi containing fluid were opened up, the fluid becoming more turbid as the pelvis was approached. Deep in the pelvis a large abscess was opened, containing thick stinking pus, which surrounded the uterus and adnexa. Forceps were passed through the abdominal opening and pushed behind the uterus in Douglas's pouch, so as to cause a bulging in the vagina, where an opening was then made which permitted the passage of a large tube, about sixteen inches long, so that one end emerged at the vulva and the other at the abdominal incision. The pelvis was sponged out, but no irrigation was employed until five days later. For some days the patient appeared scarcely likely to recover, but in the course of a week a distinct improvement occurred, in spite of the formation of a fæcal fistula. The escape of fæces ceased spontaneously, the abscess cavity gradually closed, and the patient made a good recovery, though it subsequently became necessary to open up a small sinus caused by the drainage tube in the posterior vaginal fornix.

**Dermoid tumours**—and adeno-cystomata—of the ovary are very uncommon at 15 years of age, as in Case 47, which is the earliest age at which I have been called upon to operate for these conditions. Case 47 is also interesting because she was sister to Case 29 (who was only 20 years of age when I removed an ovarian adeno-cystoma), and because dermoid and adeno-cystomatous tumours existed separately in the same ovary.

CASE 47.—**Dermoid tumour and adeno-cystoma of left ovary.**—The patient had menstruated regularly for two years. Gradual enlargement of the abdomen had been noticed for twelve months. In the left iliac fossa, close to the anterior iliac spines, a rounded elastic swelling the size of an orange could be felt, and apparently connected with this was another fluctuating swelling, which reached to just above the level of the umbilicus and to the outer edge of the right rectus. On opening the abdomen in the median line, a large multilocular cyst presented, which when tapped was found to contain a white glairy fluid (220 ozs., neutral reaction, sp. gr. 1014). Attached to this was the "lump" which was felt before operation in the left iliac fossa, and this proved to be a dermoid tumour containing a yellowish-brown grumous fluid (28 ozs., neutral reaction, sp. gr. 1006), a good deal of long light hair (much the same colour as that of the patient's head), and a large plate of bone, about two inches long and one inch broad, bearing some teeth and what appeared to be tooth-sacs attached by pedicles. The parovarium was unaffected. The tumours and the left uterine appendage were removed, after tying with an interlocking stitch. Two small cysts in the right ovary were snipped and a portion of their walls cut away, the ovary being conserved. Recovery was uneventful.

### B.—Hystero-myomectomy for Myo-fibroma of the Uterus.—Cases 25 and 49.

When the conservative operation of myomectomy is unsuitable, and it is necessary to remove uterine fibroids by hystero-myomectomy, the "retro-peritoneal" treatment of the pedicle has many self-evident advantages over the older "extra-peritoneal" method, which is now rarely used. In myo-fibromata the uterus may be amputated at the supra-vaginal cervix: it is unnecessary to remove the whole uterus, as would be done in cases of carcinoma. There are several ways of performing this operation, but one of the simplest methods is that which was employed in the following example :—

Case 25.—**Myo-fibroma uteri—Retro-peritoneal hystero-myomectomy.**—A gradual enlargement of the abdomen had been noticed for about four years. There was great pain—almost constant—in the back and groins, and micturition was frequent. Menstruation had latterly been excessive, and the patient was steadily losing ground. The lower abdomen was occupied by a firm, nodular, globular tumour in the mid-line, rising out of the pelvis and reaching above the umbilicus. *Per vaginam*, this was felt to be part of a generally enlarged uterus, the right broad ligament being considerably involved in the growth. An incision four and half inches long was made in the median line, between the umbilicus and pubes. On opening the peritoneum the tumour presented in the middle line, and both ovaries were found to be enlarged—the right ovary being also cystic. The Trendelenburg posture was then adopted, and the tumour delivered through the parietal wound by means of a myoma screw. The right ovarian vessels were tied in two places, close to the pelvis, beyond the fimbriated extremity of the Fallopian tube, and the tissues between the ligatures divided. The right round ligament was tied in two places and divided between the ligatures. The left ovarian vessels and round ligament were then similarly tied and divided. In this way the broad ligament on either side was opened up. The incisions dividing the round ligaments were then joined by an incision extending across the anterior surface of the tumour, situated about two-thirds of the way down from the summit of the tumour to the supra-vaginal cervix. This transverse incision involved the peritoneum only, and by peeling downwards the peritoneal flap so formed the bladder was freely separated from the tumour. On passing the finger down into the connective tissue at the sides of the uterus the uterine arteries could be felt pulsating, and these were tied on either side by means of silk carried under the vessels with curved pedicle forceps. An incision was then made through the peritoneum on the posterior surface of the tumour. The ends of this incision joined that on the anterior surface, so as to encircle the tumour. A posterior flap of peritoneum was then formed by peeling this structure off the tumour, as in the formation of the anterior peritoneal flap. The tumour was amputated by cutting across it just above the top of the supra-vaginal cervix. The amputation was made in a cup-shaped fashion, so as to leave a concave stump. A second ligature was placed on each ovarian and uterine vessel, and the anterior and posterior flaps of peritoneum were united over the stump by means of a continuous silk suture. The parietal wound was completely closed. The wound was dressed three times at intervals of a week, the stitches being removed at the last dressing when the patient was able to walk about.

### C.—**Operation for Hepatic Abscess.**—Case 49.

I have elsewhere[1] referred to the desirability of treating cases of hepatic abscess and of hydatid of the liver by direct hepatotomy rather than by operation *à deux temps*. The majority of liver abscesses tend to cause enlargement of that organ downwards, so that direct hepatotomy can be carried out by the ordinary anterior abdominal incision. In some cases, however, the abscess tends to encroach upon the thorax, and then the most direct route for purposes of evacuation is across the pleural cavity. This method of opening hepatic abscesses is exemplified in the following case:

Case 49.—**Hepatic abscess—Trans-pleural hepatotomy.**—The patient had lived in India for six years, and there contracted ague six years, and typhoid fever two years, before the present illness. Five weeks before he came under observation he "caught cold," and was seized with griping pains in the abdomen. A swelling on the right side of the abdomen developed, and there was progressive emaciation and weakness. He looked profoundly ill, with sallow complexion but no jaundice. There was a considerable bulging and much tenderness over the whole liver area, and a coarse friction could be heard in the right axillary line of the lower part of the thorax and along the neighbouring costal margin. Dulness extended up the back to nearly half-way up the scapula, but breath sounds were clearly heard and there was no diminution of vocal fremitus or vocal resonance. An incision was made along the ninth rib, half-way between the spine and sternum, and about two and a half inches of the rib were excised. The costal pleura was opened, so as to expose the pleura covering the diaphragm. The edges of the incision in the costal pleura were then sewn down to the pleura covering the diaphragm, so as to include a circle of the latter about one and a quarter inches in diameter. By this means the pleural cavity was entirely shut off from the area of operation enclosed by the continuous silk stitch. An incision was then made through the diaphragm into the liver beyond, and thirty-two ounces of thick yellow pus were evacuated from a smooth-walled cavity in the liver which could not be entirely explored. A large drainage tube was placed in the cavity, which was irrigated daily after the patient had begun to gain strength. A bacteriological examination

[1] *Bristol M.-Chir. J.*, 1898, xvi. 222; and *Brit. M. J.*, 1898, ii. 149.

of the pus proved it to be sterile. The right lung—which necessarily collapsed on opening the pleura—soon expanded, and rapid improvement took place, but the track of the drainage tube did not finally close until some months afterwards.

### D.—**Operations on the Gall-Bladder.**—Cases 8, 21, 22, and 40.

The diagnosis of cases attended with jaundice is often a matter of difficulty. In Case 8, a stone in the common duct was regarded as the probable cause of the infective cholangitis, especially as the gall-bladder was not enlarged; but in Case 21, malignant disease was suspected because of the distension of the gall-bladder which existed. In neither case, however, could a diagnosis be definitely made, and an operation which was regarded as mainly exploratory was quite justifiable. In each case the operation terminated in cholecystostomy with the object of preventing the effects of **cholæmia**, from which the patients were clearly suffering. This was of temporary benefit in Case 8, but resulted in death from secondary hemorrhage in Case 21. This tendency of operation to cause death from hemorrhage in patients suffering from jaundice is well known, and Mayo Robson has suggested that an attempt should be made to prevent it by the administration of chloride of calcium for some days before operation. This was done in both these cases. It is not generally recognised that all cases of **enlarged gall-bladder** without jaundice, supposed to be due to cholelithiasis, should be subjected to operation whether there is pain or not. One of the dangers of delay (acute cholecystitis) is exemplified in Case 22, which would certainly have had a fatal termination without an operation which is better performed to prevent, rather than to cure, such mischief as gallstones are capable of producing (*vide infra*). Case 40 was an interesting one of **catarrhal cholecystitis** (hydrops), apparently due to kinking of the cystic duct from adhesions. Cholecystostomy resulted in complete cure. The case has been described fully elsewhere.[1]

Case 22.—**Acute cholecystitis — Cholecystostomy.** — A swelling had existed in the situation of the gall-bladder for about twenty years, but it remained stationary and gave no trouble till three weeks before the patient came under my observation, when it began to enlarge and become tender. The swelling increased, and a succession of rigors

[1] *Brit. M. J.*, 1900, i. 1329.

occurred, the temperature rising to 104° or 105°. There was marked general prostration; no jaundice was present. To the right of the umbilicus a distinct bulging of the abdominal wall could be seen, caused by a smooth globular tumour nearly the size of a cricket-ball, moving with respiration and feeling like a tense cyst. This swelling was movable laterally, but could not be displaced downwards because of an apparent connection with the liver. The liver was slightly enlarged, and a distinct sulcus could be felt between this organ and the swelling below it. A vertical incision over the globular swelling exposed a linguiform process (Riedel's lobe) of the liver, and beyond this was seen a distended gall-bladder the size of a goose's egg. Nine ounces of thin pus were removed by aspiration from the gall-bladder, which was then incised and twenty-eight gallstones removed. Of these, eight were as large as small hazel nuts, the rest much smaller. One stone was impacted in the cystic duct, and directly this was dislodged bile flowed into the gall-bladder. The opening in the gall-bladder was sutured to the aponeurosis in the ordinary way, and a drainage tube was kept in for a week. Two ounces of bile came daily through the wound at first; but the flow entirely ceased in the course of three weeks, and the fistula healed soon after. A culture made from the pus removed from the gall-bladder at the time of operation showed short motile bacilli, forming gas in glucose, agar, &c., which were probably bacilli coli communes. (Dr. Symes.)

The linguiform process of liver above referred to is generally found in association with cholelithiasis, but I have seen it in cases in which no gallstones were present. Probably any condition which causes adhesions between the gall-bladder and the liver may lead to elongation of a portion of the adherent liver in association with an enlargement of the gall-bladder. Of such causes, gallstones are, doubtless, the most common.

### E.—**Operation for Tubercular Peritonitis.**—Case 18.

Cœliotomy holds out a very fair prospect of cure in the subacute and chronic forms of the "ascitic" variety, and in many cases of the "fibrous" and "ulcerous" varieties of tubercular peritonitis.

Acute miliary tuberculosis of the peritoneum is not suitable for operation. Such disease is liable to be confused with appendicitis, as in a case described in my former paper.[1] The resemblance to appendicitis was very marked also in the following case of subacute tubercular peritonitis; and I could not be sure, even after operation, that the lesion found had not actually spread from a tubercular inflammation of the appendix.

[1] *Bristol M.-Chir. J.*, 1898, xvi. 219.

CASE 18.—**Tubercular peritonitis—Cœliotomy.**—The illness began four weeks before operation, with marked pain in the right iliac fossa, especially before and during defæcation. The bowels were loose and had been streaked with blood. The temperature kept at about 101°. There was no tubercular family history. Just internal to the right anterior superior iliac spine an indurated area could be felt reaching inwards for some two and a half inches from the iliac spine and shading off along Poupart's ligament. This swelling was fixed and tender. On examination *per rectum*, some matting could be felt in the pelvis. An incision made near the right anterior iliac spine disclosed a large amount of tubercular granulation tissue in the ileo-cæcal region, matting the intestines in the neighbourhood together. Numerous mesenteric glands were found enlarged, and the intestines generally were covered with miliary tubercles. The granulation tissue was freely scraped away and the abdomen closed. The temperature was normal after the fourth day from the time of operation, and there was a gradual gain of flesh and strength. After a visit to Weston, during convalescence, the child continued quite well.

### F.—**Operation for Peritoneal Adhesions.**—CASE 1.

Almost any inflammatory trouble within the abdomen may give rise to adhesions which may produce symptoms varying from slight dragging pain to intestinal obstruction. Considering the large number of abdominal sections which is now performed, it is remarkable to find how rarely we are called upon to operate for adhesions consequent upon a cœliotomy.

CASE 1.—**Peritoneal adhesions.**—The patient had been subjected four years previously to right salpingo-oöphorectomy—the right uterine appendages being found attached to the sigmoid flexure. Six weeks after this operation she complained of hypogastric pain (both sides), which had gradually increased in intensity. Cœliotomy disclosed numerous adhesions between the great omentum and the intestines, forming a series of loculi. The adhesions were liberated and the patient was entirely relieved of her pain.

### G.—**Operations on the Intestines.**—CASES 2, 15, 19, 26, 33, 36, 38, 43, 46, and 48.

With two exceptions—cases 33 and 48—these operations were performed for malignant disease of the intestines.

The operations might be subdivided as follows:—

**Sigmoidostomy and Colostomy.**—CASES 2, 36, 38, and 46.

**Colectomy.**—CASES 19, 26, and 43.

**Enterostomy.**—CASES 33 and 48.

**Ileo-colostomy ("short-circuiting").**—CASE 15.

The continued multiplication of methods for uniting divided intestine bears witness to the unsatisfactory condition of this branch of abdominal surgery. Much of the want of success in these cases is due to circumstances which have already been dwelt upon in the opening remarks. In the performance of **intestinal resection,** I always prefer the use of simple suture to that of mechanical appliances, and of all intestinal sutures (and their number is large) I know none better or more generally applicable than Halsted's quilt suture. In early cases of **malignant disease** of the intestine, the ideal method of treatment is that of resection and simple suture of the divided ends; but in cases where the patient is already suffering from symptoms of obstruction it is best, whenever possible, to bring the growth to the surface, open the intestine above the growth to relieve the over-distended bowels of their contents, and subsequently to remove the growth and unite the two ends of the divided intestine. This is the method advocated by Greig Smith, and was practised with success in Case 19.

In malignant disease of the ascending (Case 43) and descending (Case 26) colon, it is frequently impossible to bring the growth to the surface, and here one often has to be content with union of the gut by some mechanical device after resection of the involved segment. For this reason a Mayo Robson's bobbin was used in Case 26, and a Murphy's button was used in Case 43, but both patients died from faults associated with these appliances—the bone bobbin was too soft and collapsed when the sutures were tied, so that leakage occurred, and the Murphy's button caused a pressure gangrene of the bowel beyond the limits of the button.

**Sigmoidostomy and colostomy** in irremovable malignant disease are best performed by passing a glass rod through the mesocolon after the bowel has been brought to the surface (Reclus's method). Such operations are, however, poor substitutes for the efficient help which surgery can often afford in an earlier stage of the disease. Truly the patient is saved from death from intestinal obstruction, but the comparatively short prolongation of life which the operation gives is hampered by the inconvenience of an inguinal anus.

Whenever possible, a **short circuit** should be made in preference to the formation of an artificial anus. The following is an example of this operation in a case of malignant disease of the ascending colon :—

Case 15.—**Short circuiting** (**Ileo-colostomy**).—An incision over the right side of the abdomen, one and a half inches internal to the anterior superior iliac spine, showed the cæcum and ileum to be enormously distended and congested. The former was as large as a fœtal head at full term, and the longitudinal muscular bands stood out like large cords, half an inch wide. The ileum was about three and a half inches in diameter, and the vermiform appendix was the size of ordinary small intestine. In the ascending colon, about two and a half inches below the ileo-cæcal valve was a hard, irregular mass, about as large as a fist, which had apparently almost closed the lumen of the gut. The fixity of the growth, and the existence of numerous hard enlarged glands in the neighbourhood and secondary deposits in the anterior wall of the cæcum, rendered resection impossible. Short circuiting was therefore performed by uniting the ileum (about six inches from the ileo-cæcal valve) to the transverse colon (just beyond the hepatic flexure) by Halsted's method of suture. The opening between the two portions of gut was about one and a half inches long. The transverse colon was no larger in diameter than the thumb. The bowels acted daily after operation, until the patient's death occurred from gangrenous pneumonia.

### H.—**Exploratory Operations.**—Cases 3, 6, 7, 23, 35 and 50.

Most of these operations were performed for **malignant disease** of the gastro-intestinal tract. The diagnosis was correctly made before operation in all the malignant cases, but cœliotomy was performed because it was impossible to be sure until the abdominal cavity was opened whether the growth was removable or not.

Not one of this group was injuriously affected by the operation, and the low mortality of exploratory operations of this kind fully justifies the procedure in cases of uncertain knowledge, either of the nature or extent of the disease. When this fact is more fully appreciated, we shall have a better opportunity of treating malignant disease of the abdominal viscera in an early stage.

Case 7 was a **retroperitoneal lipoma** nearly as large as an adult's head, which simulated an ovarian cyst. There was, however, resonance over the pubes, which rarely obtains in cases of ovarian tumours. The attachments of the lipoma were so extensive that no attempt was made to remove it.

J.—**Operations for Appendicitis.**—CASES 4, 9, 14, 16, 17, 28, 30, 32, 42, 44 and 45.

In cases of **suppurative appendicitis**, the safest rule is to remove the appendix in those cases only where it is easily reached, and where it does not form a part of the adhesions which may help to shut off the abscess cavity from the general peritoneal cavity. In other words, it is removed in that class of appendicitis where the appendix floats freely in a cavity surrounded by matted coils of intestine which have to be separated in order to liberate the pus.

**Appendicectomy** was performed in Cases 9, 14, 16, 28, 30, 32, 42, and 45; but of these, Cases 16, 30, and 42 were **relapsing cases**, and the appendix was removed in an interval between the attacks, and Case 9 was removed for a **persistent sinus** communicating with the appendix in a case which had been previously treated by drainage only.

Secondary operations of this last class are by no means common, and it is probable that in most cases of suppurative appendicitis treated by drainage only, the appendix becomes atrophied,—it certainly gives rise to further trouble with comparative rarity.

In this series there were three deaths—Cases 4, 14, and 28. Of these, Case 4 was associated with diffuse peritonitis, and operation was practically only a forlorn hope; Case 14 was one of gangrenous appendicitis with diffuse peritonitis, and Case 28 developed cerebral thrombosis and died after a series of epileptiform convulsions.

Case 30 was interesting because the appendicitis was associated with a chain of enlarged calcareous glands which were removed from the meso-cæcum and meso-colon. It is rare for **gangrenous appendicitis** to give rise to other than the most acute symptoms, which generally end fatally from diffuse peritonitis, but in the following case a localised abscess formed and the peritonitis was strictly limited:—

CASE 45.—**Gangrenous appendicitis.**—Ten days before operation the attack began with pain near the umbilicus (subsequently limited to the right iliac fossa), vomiting, and temperature of 102°. A swelling gradually formed in the right iliac fossa, which soon began to diminish in size, but it had nearly doubled in size during the preceding fortyeight hours, and at the time of operation was nearly as large as the fist and occupied a position along the outer half of Poupart's ligament. An incision over the tumour and separation of a few adhesions disclosed a cavity containing about one and a half ounces of stinking pus. The appendix which was lying along the wall of the cavity was easily separated, and found to be entirely gangrenous, so that it broke away at its cæcal attachment. Recovery was uninterrupted.

I have practically discarded **irrigation of the peritoneal cavity,** and it was not employed in any of the above cases of appendicitis. Irrigation, by the conveyance of infected material to unaffected parts, is productive of much harm, and more cases of suppurative appendicitis will be saved by simply mopping out the affected area than by attempts to "cleanse" the peritoneum by means of a douche.

### CASES OF ACUTE INTESTINAL OBSTRUCTION TREATED BY OPERATION.[1]

BY

W. H. HARSANT, F.R.C.S.,

